# Controlled testing of novel portion control plate produces smaller self-selected portion sizes compared to regular dinner plate

**DOI:** 10.1186/s40608-017-0167-z

**Published:** 2017-07-28

**Authors:** Joel W. Hughes, Carly M. Goldstein, Carly Logan, Jessica L. Mulvany, Misty A. W. Hawkins, Amy F. Sato, John Gunstad

**Affiliations:** 1 Department of Psychological Sciences, Kent State University, P.O. Box 5190, Kent, Ohio 44242 USA; 2 Department of Psychiatry and Human Behavior, Brown University, Providence, Rhode Island USA; 3 Department of Psychology, University of Florida, Gainesville, Florida USA; 4 Department of Psychology, Oklahoma State University, Stillwater, Oklahoma USA

**Keywords:** Portion size, Obesity, Overweight, Energy intake, Cognitive science, Optical illusions

## Abstract

**Background:**

Obesity is a global health crisis, and portion control is a key method for reducing excess body weight. Given consumers’ familiarity with large portion sizes, reducing portion sizes can be difficult. Smaller plates are often recommended to reduce portion sizes and appear to reduce portion sizes. However, there are no studies evaluating dishes specifically designed to facilitate portion control. The aim of the present study was to validate the efficacy of a novel portion control plate inspired by the Ebbinghaus and Delboeuf visual illusions to promote serving smaller portions compared to a larger dinner plate.

**Methods:**

In two studies with a total of 110 university students, we determined whether the use of the portion control plate would result in smaller food portions compared to a larger dinner plate. The portion control plate was smaller and incorporated portion size indicators. Study 1 used instructions from My Plate based on plate ratios (e.g., “the USDA recommends filling half your plate with vegetables”) and study 2 used absolute portion size recommendations (e.g., “1 cup of vegetables”).

**Results:**

The portion control plate produced smaller self-selected servings in both studies. However, the servings of vegetables selected were smaller than recommended portion sizes for both the portion control plate and the regular dinner plate.

**Conclusions:**

Portion control plates have the potential to reduce self-selected portion sizes. Future research should include studies in a broader range of ages and clinical trials of portion control dishes for weight loss.

**Electronic supplementary material:**

The online version of this article (doi:10.1186/s40608-017-0167-z) contains supplementary material, which is available to authorized users.

## Background

**Fig. 1:**
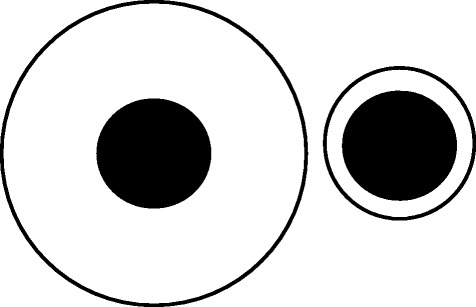
Delboeuf Illusion. Permission to use this figure was not required because this figure was created by the lead author

Obesity has been declared an epidemic by the World Health Organization [1, 2] as nearly 35% of individuals in the United States are obese [3] and the worldwide prevalence of obesity has risen to 36.9% for men and 38% for women [4]. One reason for the obesity epidemic is the excess intake of energy combined with reduced energy expenditure, with large portion sizes implicated as a key player in promoting this imbalance [5]. Many food portions sold in the United States exceed the United States Department of Agriculture (USDA) and Food and Drug Administration (FDA) standards by alarming proportions [6]. For example, typical cookies average 700% larger than USDA standards, cooked pasta 480% larger, steaks 224% larger, and bagels 195% larger [7]. As might be expected, portion control strategies are commonly recommended [8] and were emphasized in the 2010 and 2015 USDA dietary guidelines [4, 9]. Unfortunately, many persons have difficulty learning the healthy portion sizes for different foods and consistently consuming those amounts [10]. Accordingly, the need for tools to enhance portion control is clear and has inspired investigations of the influence of serving dishes and plates design on portion size and food consumption. Based on the Delboeuf illusion (Fig. 1; [11, 12]), in which a similar amount of food looks larger on a smaller vs. larger plate, the recommendation to use smaller plates is now widespread.

The recommendation to use smaller plates appears supported by a recent meta-analysis which concluded that larger plate sizes result in greater self-selected portion sizes and food consumed, but that plate size did not affect consumption of fixed portion sizes from different sized plates [13]. Furthermore, the effect of plate size was stronger when people were not aware that they were in a food study, such as when they were distracted with other tasks or participating under a cover story [13, 14]. Another systematic review concluded that dishware size does not have a consistent effect on food intake. [15] However, there is little research on the intentional use of portion control dishware.

We reasoned that people using portion control dishes would be attempting to reduce portion sizes to reduce caloric intake. For example, a randomised clinical trial employing a commercially available portion control plate and bowl (The Diet Plate, Glossop, England [16]) in a weight loss intervention for patients with type 2 diabetes reported greater weight loss in the group using the portion control dishware than in the treatment-as-usual control group [17]. In this trial the portion control dishware was not merely smaller than comparison dishes, but instead had portion size indicators for common foods.

**Fig. 2:**
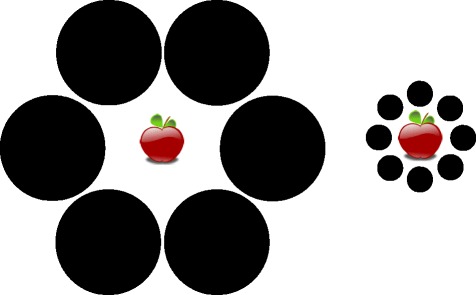
Ebbinghaus Illusion. Permission to use this figure was not required because this figure was created by the lead author

**Fig. 3:**
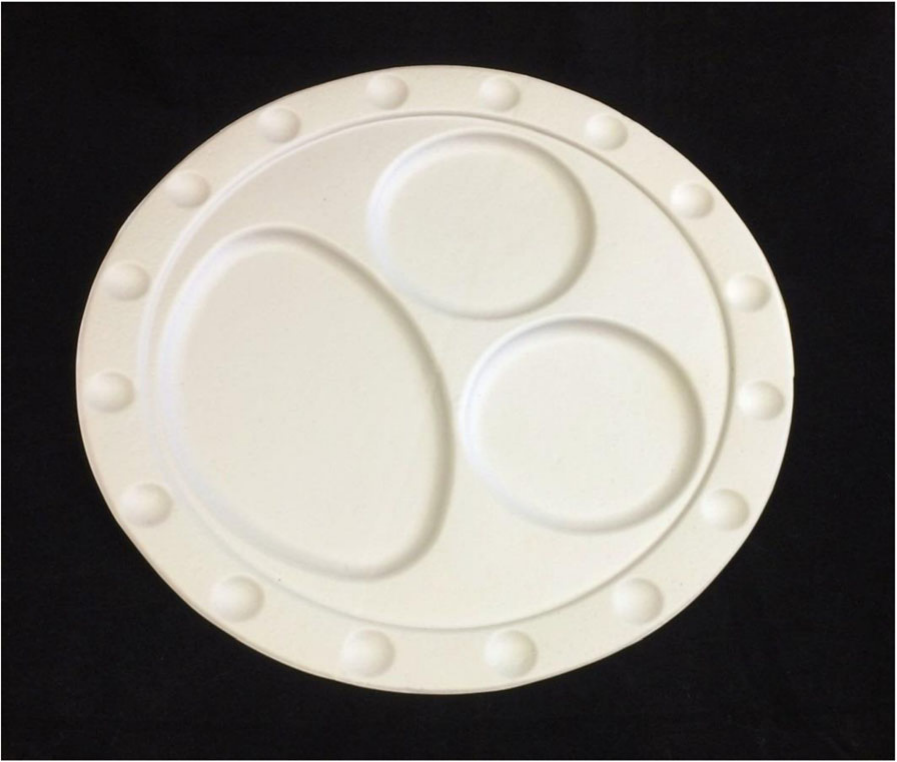
Portion control plate inspired by the Delboeuf and Ebbinghaus Illusions

Portion control dishes designed to incorporate both portion size indicators and visual illusions from the cognitive science literature might be effective in reducing portion sizes. The senior authors (JWH and JG) designed a plate inspired by the Delboeuf illusion (Fig. 1; [11, 18]) and the Ebbinghaus illusion (Fig. 2; [19]). In the Delboeuf illusion, the two inner solid “circles” (represented by apples in Fig. 2) are the same size. In the Ebbinghaus illusion, a circle appears larger if it is surrounded by smaller circles. We also added portion size indicators consistent with standard portion sizes (in the USA) for fruit or vegetables (1 cup/236.6 ml), grains or starches (1/2 cup/118.3 ml), and protein (3 oz./85 g) [4, 9] (see Fig. 3). The intent was to create a plate that facilitates selecting appropriate portion sizes on a relatively small plate. Here we report the initial validation experiments using this design.

We tested the efficacy of a portion control plate designed to promote the serving and consumption of smaller portions. In two studies, we determined whether use of the portion control plate would result in selection of smaller portions compared to a large dinner plate. We hypothesized that participants would place less food onto the portion control plate compared to the comparison plate. Study 1 used instructions based on plate ratios from myplate.gov (e.g., “the USDA recommends filling half your plate with fruits or vegetables.”). Study 2 used instructions based on serving sizes (e.g., “1 cup fruits or vegetables”). Study 2 was conducted because the instructions given in Study 1 could result in exaggerated differences between portion sizes for the different plates. That is, the portion sizes participants were instructed to select were relative in Study 1 (“1/2 the plate”) vs absolute in Study 2 (“1 cup”).

## Methods

### Design

Both studies used a 2 (plate: portion control versus comparison) × 2 (order: portion control first versus portion control second) design. All participants used both plates in order to increase power by controlling individual differences (e.g., hunger, food serving habits). A delay of 10 min elapsed between conditions, and the order of plate presentation was counterbalanced across subjects.

### Materials

**Fig. 4:**
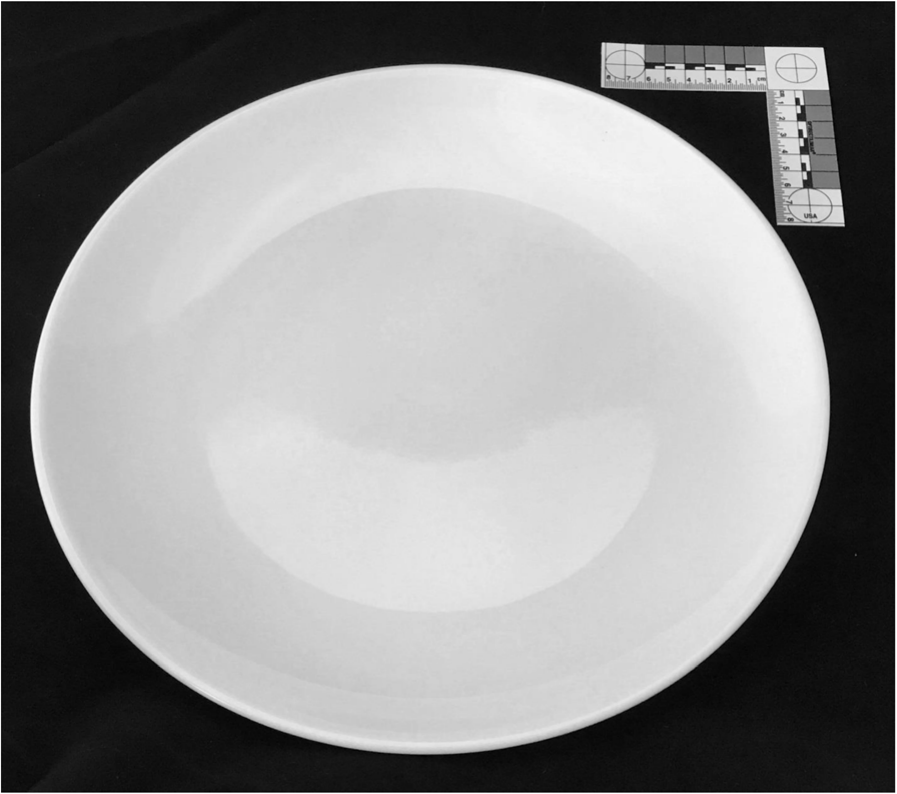
Large comparison plate

A portion control plate was designed for the present study by the senior authors (JWH and JG) and prototyped using vacuum forming from food-grade plastic material (see Fig. 3). This plate combines the Delboeuf and Ebbinghaus illusions in an attempt to increase the perceived portion size of food served. The plate measures 25 cm in diameter (area = 490.87 cm^2^), and has a border of 2 cm leaving a 21 cm diameter area for plating food. The plate also has serving size indicators for grains/starches, protein, and fruit/vegetables, which reduce the usable plating area. The protein and grains circles are 8.5 cm in diameter and .5 cm deep. The fruit/vegetable oval was 14.5 cm by 9.5 cm. For comparison, large white porcelain dinner plates were purchased from an online retailer [20]. The plate chosen was a 30.48 cm diameter (area = 729.7 cm^2^) white porcelain plate (Model COP-21 by CAC China [21]) and had no distinguishing markings (e.g., border, edge) (see Fig. 4).

Three slow cookers with a 4.7 l capacity were used to prepare protein, grain and vegetables to be portioned onto the plates by participants. The protein chosen was Tyson^®^ Grilled & Ready^®^ Chicken Breast Strips (Tyson, Inc., Springdale, AR, USA). The grain was FoodClub^®^ enriched long grain rice (Topco Holdings, Inc., Topco Associates LLC., Elk Grove Village, IL, USA). The vegetables were FoodClub^®^ canned sweet peas (Topco Holdings, Inc., Topco Associates LLC., Elk Grove Village, IL, USA). The slow cookers were approximately 80% full and maintained at a uniform level of fullness for all study participants.

### Participants and procedures

Participants were recruited through a psychology department research recruitment system as well as from poster advertisements distributed across the campus of a large, Midwestern university in the United States. Few inclusion and exclusion criteria were specified; participants were English-speaking university students and were asked not to participate if they reported intolerance or allergy to the foods used (although they were not consumed). Study 1 included 70 university undergraduates (47 females and 23 males) aged 18 to 33 who participated in the first study (see Table 1). Study 2 included 40 undergraduates (29 females and 11 males) aged 18–30 (see Table 2). No individuals participated in both studies.

**Table 1:** Characteristics of Participants in Study 1 (*N* = 70)

	*M*(*SD*) or *N*(%)
*Demographic Factors*	
Age	20.0(2.9)
Male	23(33)
Hispanic	3(4.3)
Minority	14(20.0)
University Education Level	
First year	36(51.4)
Second year	10(14.3)
Third year	11(15.7)
Fourth year	13(18.5)
BMI	25.4(5.8)

**Table 2:** Characteristics of Participants in Study 2 (*N* = 40)

	*M*(*SD*) or *N*(%)
*Demographic Factors*	
Age	20.0(2.0)
Male	11(27.5)
Hispanic	2(5.0)
Minority	7(17.5)
University Education Level	
First year	9(22.5)
Second year	8(20.0)
Third year	11(27.5)
Fourth year	11(27.5)
Post-baccalaureate	1(2.5)
BMI	25.1(5.0)

Both studies took place in a university psychology laboratory setting. Participants completed the procedures individually and used the plates in a private room that did not contain any measuring devices (e.g., scale). Participants provided informed consent and completed a demographics form. The participants were then presented with the first plate trial. The type of plate they received first (portion control plate or comparison dinner plate) was randomized. For study 1, participants were instructed that “the USDA recommends filling half your plate with fruits or vegetables, one quarter of your plate protein, and one quarter of your plate grains,” consistent with USDA guidelines [4] and the MyPlate [22, 23] guidelines. For study 2, participants were instructed to select amounts consistent with one portion of each of three types of food: “3 ounces of protein, 1 cup of vegetables, and one half cup of grains.” When giving instructions for the portion control plate, researchers pointed to each of the serving size indicators. After serving food onto the first plate, participants completed self-report questionnaires for 10 min as a distractor task. The questionnaires included The Penn State Worry Questionnaire [24], a questionnaire that inquired about texting and driving, and a questionnaire that asked questions about smartphone application usage. After working on the distractor items for 10 min, the participants were presented with the alternate plate and were given the same instructions for portioning food onto the plate. Out of sight of the participants, the food that had been served onto the plates was then weighed in grams. Height, weight, and BMI were recorded for each subject following portioning out the food onto both plates (see Tables 1 and 2). At the end of the study, subjects were compensated and debriefed.

### Measures

An Ohaus Scout Pro SP2001 electronic scale was used to weigh the food to the hundredth gram after each trial (Parsippany, NJ, USA). A demographics and health behavior survey form was used to record information on age, gender, height, weight, years of education, employment type/status, race, and the presence of any chronic medical conditions. Height and weight were measured after other study procedures using a Detecto model 439 balance scale with height bar (Detecto Scale, Webb City, MO, USA).

### Data analysis

All analyses were performed using IBM SPSS (version 22, IBM Corporation, Armonk, NY, USA). Multiple 2 (gender: male versus female) × 2 (plate: portion control versus comparison) × 2 (order: portion control plate first versus second) analyses of variance were conducted to compare portion sizes in grams. Plate was a repeated measures variable. Effects with *p*< .05 were considered significant. We did not correct for multiple comparisons because we had directional hypotheses for three effects. Also, any effects of gender or order, as well as any interactions, would not necessarily support our hypotheses. Therefore, we chose a less stringent significance level in order to detect these potential effects. Simple main effects were performed for any significant interactions observed. Sample sizes were chosen a priori and no interim analyses were conducted. The results of Study 1 were not known before Study 2 was completed. Results were summarized and presented in figures for conciseness, but all cell means are presented in the Additional file 1.

## Results

### Portion sizes in study 1

**Fig. 5:**
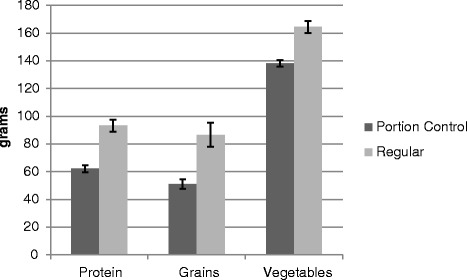
Portion sizes in study 1 by plate and food type

Results for Study 1 are summarized in Fig. 5. For protein, portion size was significantly greater on the regular plate (*M* = 93.3 g, *SD* = 35.3) compared to the portion control plate (*M* = 62.1 g, *SD* = 20.6), *F* (1, 66) = 59.3, *p*< .0005. No other main effects or interactions were observed, *p*’s > .15. For grains, a three-way interaction of gender, order, and plate type was observed, *F* (1, 66) = 6.30, *p* = .02. Simple main effects were performed by conducting analyses separately for the two orders of plate presentation. When the portion control plate was presented first, portion size was larger on the regular plate compared to the portion control plate, *F* (1,33) = 31.82, *p*< .0005. When the portion control plate was presented second, main effects of gender (*p* = .01) and plate type (*p*< .005) were moderated by an interaction of gender with plate type, *F* (1, 33) = 4.87, *p* = .04. Examination of marginal means suggested that men presented with the regular dinner plate first served larger portions of grains (*M* = 115.9 g, *SD* = 28.2) than women presented with the regular dinner plate (*M* = 79.5 g, *SD* = 39.3), whereas the portion sizes for the portion control plate were more similar between men (*M* = 58.8 g, *SD* = 24.1) and women (*M* = 49.8 g, *SD* = 22.4).

For vegetables, an interaction of order and plate type was observed, *F*(1,66) = 4.87, *p* = .031. When the portion control plate was presented first, participants placed larger portions on the regular plate (*M* = 164.5 g, *SD* = 64.3) than the portion control plate (*M* = 83.3 g, *SD* = 29.2), *F*(1,33) = 7.0, *p* = .01. When the regular plate was presented first, participants again placed larger portions on the regular plate (*M* = 138.3 g, *SD* = 80.1) than the portion control plate (*M* = 98.0 g, *SD* = 29.0), *F* (1,33) = 6.9, *p* = .01. The interaction appears to have been due to a smaller effect when the regular plate was presented first.

### Portion sizes in study 2

**Fig. 6:**
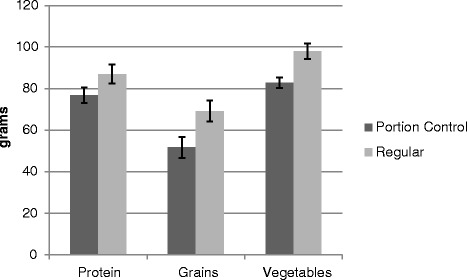
Portion sizes in study 2 by plate and food type

Results for Study 2 are summarized in Fig. 6. Effects of plate type were observed for protein, *F* (1, 36) = 6.9, *p* = .01, vegetables *F* (1, 36) = 10.58, *p* = .002, and grains, *F* (1, 36) = 14.55, *p* = .001. For each food, portion sizes were larger for the regular plate compared to the portion control plate. No order effects, gender effects, or interactions were observed.

## Discussion

The findings of the study indicate that use of a portion control plate resulted in smaller portion sizes for protein, starch, and vegetables. In Study 1, interactions notwithstanding, participants served smaller portions (33% less chicken, 41% less rice, and 16% less peas) on the portion control plate than the regular dinner plate. In Study 2, participants served smaller portions (12% less chicken, 25% less rice, and 16% less peas) on the portion control plate than the regular dinner plate. Instructions based on both MyPlate (e.g., “half vegetables”) and USDA portion sizes (e.g., “one cup vegetables”) yielded similar results.

In previous studies, the differences between smaller and larger plates ranged from 30% larger to 200% larger in the studies included in a recent meta-analysis [13]. In the studies reported here, the area of the portion control plate was 33% smaller than the regular plate. The effect size observed in this literature depends on several factors, but halving the plate size was suggested to result in roughly a 29% reduction in self-served portions [13], which compares favorably with the reductions in protein and starch we observed.

Portion control dishes used intentionally may yield stronger effects than merely switching from larger to smaller plates, as there are negative reports of the effects of plate size on portion sizes. For example, a recent review of the effects of dish size on self-selected portion size argued that the results can be explained by the effects of distraction and the type of food serving dish used [14]. Specifically, studies that used a smaller container only yielded reduced food consumption when also paired with distraction (e.g., [25–27]). Furthermore, all negative findings used different sizes of plates (e.g., [28]) in contrast to the studies finding an effect which often used bowls or other containers. Moreover, a Cochrane review of tableware size for changing food consumption reported a small to medium effect of tableware size on food selection [5] and argued that reducing portion size could reduce caloric intake by 8.5–13.5%. Our study made no effort to distract participants and provided clear instructions in keeping with our assumption that consumers using portion control dishes intentionally would be trying to achieve smaller portion sizes. Our design also incorporated portion size indicators, which may encourage smaller portions than observed with smaller plates alone.

One unexpected and potentially important finding was that participants consistently underserved vegetables. Although peas can spread fairly thinly on the plate, in the first study participants served 36% of the intended serving size of vegetables on the portion control plate compared to 60% on the regular plate. Whereas a number of portion control dishes are commercially available, we are unaware of any having been empirically validated with respect to the serving sizes that users actually achieve. Consumers overestimate the amount of vegetables that they serve themselves [29]. Exacerbating the under-serving of healthy foods like grains and vegetables could be an unintended consequence of portion control dishware, a hypothesis that deserves further study. Reducing vegetable consumption would be contrary to best practices for weight control, which emphasize increased fruit and vegetable consumption, in part as a strategy to reduce the energy density of food eaten [9]. Although none of the foods in Studies 1 and 2 were particularly energy dense, in Study 1 participants using the portion control plate served themselves 97 fewer kcal on average (405.8 kJ), but only 15 were from vegetables (peas) whereas 37 were from chicken. It could be argued that reducing portion sizes of vegetables does not serve the public interest of encouraging consumers to meet the daily recommended allowance of fruits and vegetables.

### Clinical and research implications

Our findings suggest that portion control dishware may be a useful tool for controlling portion sizes. Future research should examine whether portion control dishware can contribute to weight loss. We are aware of only two randomized clinical trials of portion control dishes for weight loss, both of which reported positive results [8, 17]. In contrast, there are studies documenting the success of achieving portion control by using pre-portioned meals and liquid meal replacements [30–32]. Portion control practices are uncommon, but are more likely among women and the health conscious [33]. Portion control dishware may also be particularly well suited to interventions with children, who self-select larger portions when using adult dishes [25, 34].

### Limitations

The sample was comprised entirely college students, and future studies should be conducted with other age ranges (e.g., children and older adults). The participants were not necessarily attempting to lose weight, so results may have differed for participants motivated to choose smaller portions for weight loss. The participants did not consume the food, although recent research suggests that people would typically consume 92% of food they portion onto dishware [35]. Although our design was inspired by the Delboeuf illusion and Ebbinghaus illusion, we cannot assert that these well-known phenomena account for our findings as we did not collect any data showing that these were the mechanisms for the observed effects, nor can we definitively attribute the effects to any design features given that the portion control and comparison plates were different sizes. Finally, we acknowledge that the instructions had an effect on portion size and that although they were consistent across plate conditions, this study does not answer the question of what would happen if no instructions were provided as would occur in a more naturalistic context (e.g., buffet).

## Conclusion

Portion control strategies are commonly recommended [8] and were emphasized in the 2010 and 2015 USDA dietary guidelines [4, 9]. Unfortunately, many persons have difficulty learning the healthy portion sizes for different foods and consistently consuming those amounts [10]. Accordingly, the need for tools to enhance portion control is clear and has inspired investigations of the influence of serving dishes and plates design on portion size and food consumption. Portion control plates have the potential to reduce self-selected portion sizes, but may also result in smaller portions of vegetables than are recommended. Future research should include additional design work, validation of portion control dishes, studies in a broader range of ages, and clinical trials of portion control dishes for weight loss.

## Additional file

Additional file 1: Table S1. Protein weight (g) by plate type, gender, and order for Study 1. **Table S2**. Grains weight (g) by plate type, gender, and order for Study 1. **Table S3**. Vegetables weight (g) by plate type, gender, and order for Study 1. **Table S4**. Protein weight (g) by plate type, gender, and order for Study 2. **Table S5**. Grains weight (g) by plate type, gender, and order for Study 2. **Table S6**. Vegetables weight (g) by plate type, gender, and order for Study 2. (DOCX 37 kb)
