# Supplementary material for: Controlled testing of novel portion control plate produces smaller self-selected portion sizes compared to regular dinner plate
Source: BMC Obes. 2017 Jul 28;4:30. doi: 10.1186/s40608-017-0167-z (PMC5534105; doi:10.1186/s40608-017-0167-z)
Supplement: Additional file 1: Table S1. — Protein weight (g) by plate type, gender, and order for Study 1. Table S2. Grains weight (g) by plate type, gender, and order for Study 1. Table S3. Vegetables weight (g) by plate type, gender, and order for Study 1. Table S4. Protein weight (g) by plate type, gender, and order for Study 2. Table S5. Grains weight (g) by plate type, gender, and order for Study 2. Table S6. Vegetables weight (g) by plate type, gender, and order for Study 2. (DOCX 37 kb) [file 40608_2017_167_MOESM1_ESM.docx]

**Supplementary Materials.**

Supplementary Table 1: Protein weight (g) by plate type, gender, and order for Study 1

| **Descriptive Statistics** | | | | | |
| --- | --- | --- | --- | --- | --- |
|  | ORDER | gender | Mean | Std. Deviation | N |
| PROTEIN WEIGHT ON PORTION CONTROL PLATE | Portion control first | male | 60.02 | 20.94 | 11 |
|  |  | female | 61.59 | 14.91 | 24 |
|  |  | Total | 61.10 | 16.73 | 35 |
|  | Comparison plate first | male | 71.420 | 30.64 | 12 |
|  |  | female | 58.894 | 19.13 | 23 |
|  |  | Total | 63.19 | 24.02 | 35 |
|  | Total | male | 65.97 | 26.51 | 23 |
|  |  | female | 60.27 | 16.97 | 47 |
|  |  | Total | 62.14 | 20.57 | 70 |
| PROTEIN WEIGHT ON COMPARISON PLATE | Portion control first | male | 87.92 | 33.33 | 11 |
|  |  | female | 89.39 | 27.10 | 24 |
|  |  | Total | 88.93 | 28.70 | 35 |
|  | Comparison plate first | male | 113.73 | 38.12 | 12 |
|  |  | female | 89.26 | 40.30 | 23 |
|  |  | Total | 97.65 | 40.74 | 35 |
|  | Total | male | 101.39 | 37.49 | 23 |
|  |  | female | 89.33 | 33.82 | 47 |
|  |  | Total | 93.29 | 35.26 | 70 |

Supplementary Table 2: Grains weight (g) by plate type, gender, and order for Study 1

| **Descriptive Statistics** | | | | | |
| --- | --- | --- | --- | --- | --- |
|  | ORDER | gender | Mean | Std. Deviation | N |
| GRAINS WEIGHT ON PORTION CONTROL PLATE | Portion control first | male | 49.53 | 15.53 | 11 |
|  |  | female | 49.19 | 16.51 | 24 |
|  |  | Total | 49.29 | 15.98 | 35 |
|  | Comparison plate first | male | 58.82 | 24.14 | 12 |
|  |  | female | 49.84 | 22.45 | 23 |
|  |  | Total | 52.92 | 23.10 | 35 |
|  | Total | male | 54.38 | 20.58 | 23 |
|  |  | female | 49.51 | 19.43 | 47 |
|  |  | Total | 51.11 | 19.80 | 70 |
| GRAINS WEIGHT ON COMPARISON PLATE | Portion control first | male | 72.32 | 28.81 | 11 |
|  |  | female | 85.44 | 35.29 | 24 |
|  |  | Total | 81.32 | 33.54 | 35 |
|  | Comparison plate first | male | 115.95 | 28.23 | 12 |
|  |  | female | 79.51 | 39.26 | 23 |
|  |  | Total | 92.00 | 39.53 | 35 |
|  | Total | male | 95.08 | 35.67 | 23 |
|  |  | female | 82.54 | 37.00 | 47 |
|  |  | Total | 86.66 | 36.79 | 70 |

Supplementary Table 3: Vegetables weight (g) by plate type, gender, and order for Study 1

| **Descriptive Statistics** | | | | | |
| --- | --- | --- | --- | --- | --- |
|  | ORDER | gender | Mean | Std. Deviation | N |
| VEGETABLES WEIGHT ON PORTION CONTROL PLATE | Portion control first | male | 71.51 | 23.36 | 11 |
|  |  | female | 88.72 | 30.41 | 24 |
|  |  | Total | 83.31 | 29.19 | 35 |
|  | Comparison plate first | male | 104.20 | 35.06 | 12 |
|  |  | female | 94.78 | 25.60 | 23 |
|  |  | Total | 98.00 | 29.02 | 35 |
|  | Total | male | 88.56 | 33.78 | 23 |
|  |  | female | 91.68 | 28.02 | 47 |
|  |  | Total | 90.66 | 29.83 | 70 |
| VEGETABLES WEIGHT ON COMPARISON PLATE | Portion control first | male | 136.90 | 52.96 | 11 |
|  |  | female | 174.22 | 66.67 | 24 |
|  |  | Total | 162.49 | 64.34 | 35 |
|  | Comparison plate first | male | 128.16 | 78.18 | 12 |
|  |  | female | 143.63 | 82.37 | 23 |
|  |  | Total | 138.33 | 80.14 | 35 |
|  | Total | male | 132.34 | 65.96 | 23 |
|  |  | female | 159.24 | 75.54 | 47 |
|  |  | Total | 150.41 | 73.17 | 70 |

Supplementary Table 4: Protein weight (g) by plate type, gender, and order for Study 2

| **Descriptive Statistics** | | | | | |
| --- | --- | --- | --- | --- | --- |
|  | ORDER | gender | Mean | Std. Deviation | N |
| PROTEIN WEIGHT ON PORTION CONTROL PLATE | Portion control first | male | 86.08 | 22.83 | 4 |
|  |  | female | 72.77 | 24.28 | 16 |
|  |  | Total | 75.43 | 24.03 | 20 |
|  | Comparison plate first | male | 67.84 | 25.10 | 7 |
|  |  | female | 83.85 | 21.17 | 13 |
|  |  | Total | 78.25 | 23.30 | 20 |
|  | Total | male | 74.47 | 24.88 | 11 |
|  |  | female | 77.74 | 23.22 | 29 |
|  |  | Total | 76.84 | 23.41 | 40 |
| PROTEIN WEIGHT ON COMPARISON PLATE | Portion control first | male | 93.75 | 31.25 | 4 |
|  |  | female | 87.53 | 34.11 | 16 |
|  |  | Total | 88.77 | 32.85 | 20 |
|  | Comparison plate first | male | 76.19 | 28.39 | 7 |
|  |  | female | 90.13 | 23.42 | 13 |
|  |  | Total | 85.25 | 25.44 | 20 |
|  | Total | male | 82.57 | 29.24 | 11 |
|  |  | female | 88.69 | 29.32 | 29 |
|  |  | Total | 87.01 | 29.06 | 40 |

Supplementary Table 5: Grains weight (g) by plate type, gender, and order for Study 2

| **Descriptive Statistics** | | | | | |
| --- | --- | --- | --- | --- | --- |
|  | ORDER | gender | Mean | Std. Deviation | N |
| GRAINS WEIGHT ON PORTION CONTROL PLATE | Portion control first | male | 41.40 | 12.73 | 4 |
|  |  | female | 48.30 | 12.87 | 16 |
|  |  | Total | 46.92 | 12.82 | 20 |
|  | Comparison plate first | male | 57.47 | 19.02 | 7 |
|  |  | female | 55.77 | 17.70 | 13 |
|  |  | Total | 56.37 | 17.69 | 20 |
|  | Total | male | 51.63 | 18.21 | 11 |
|  |  | female | 51.65 | 15.41 | 29 |
|  |  | Total | 51.65 | 15.98 | 40 |
| GRAINS WEIGHT ON COMPARISON PLATE | Portion control first | male | 59.94 | 24.23 | 4 |
|  |  | female | 69.38 | 26.83 | 16 |
|  |  | Total | 67.49 | 26.00 | 20 |
|  | Comparison plate first | male | 66.66 | 14.19 | 7 |
|  |  | female | 72.88 | 25.08 | 13 |
|  |  | Total | 70.71 | 21.68 | 20 |
|  | Total | male | 64.22 | 17.56 | 11 |
|  |  | female | 70.95 | 25.66 | 29 |
|  |  | Total | 69.10 | 23.68 | 40 |

Supplementary Table 6: Vegetables weight (g) by plate type, gender, and order for Study 2

| **Descriptive Statistics** | | | | | |
| --- | --- | --- | --- | --- | --- |
|  | ORDER | gender | Mean | Std. Deviation | N |
| VEGETABLES WEIGHT ON PORTION CONTROL PLATE | Portion control first | male | 64.95 | 10.43 | 4 |
|  |  | female | 71.34 | 20.04 | 16 |
|  |  | Total | 70.06 | 18.48 | 20 |
|  | Comparison plate first | male | 99.90 | 44.00 | 7 |
|  |  | female | 93.05 | 35.50 | 13 |
|  |  | Total | 95.45 | 37.67 | 20 |
|  | Total | male | 87.19 | 38.80 | 11 |
|  |  | female | 81.07 | 29.60 | 29 |
|  |  | Total | 82.76 | 31.98 | 40 |
| VEGETABLES WEIGHT ON COMPARISON PLATE | Portion control first | male | 91.38 | 18.58 | 4 |
|  |  | female | 94.84 | 32.83 | 16 |
|  |  | Total | 94.15 | 30.12 | 20 |
|  | Comparison plate first | male | 104.27 | 44.37 | 7 |
|  |  | female | 100.48 | 29.16 | 13 |
|  |  | Total | 101.80 | 34.09 | 20 |
|  | Total | male | 99.58 | 36.43 | 11 |
|  |  | female | 97.37 | 30.82 | 29 |
|  |  | Total | 97.98 | 31.99 | 40 |
